# Causal Bayesian machine learning to assess treatment effect heterogeneity by dexamethasone dose for patients with COVID-19 and severe hypoxemia

**DOI:** 10.1038/s41598-023-33425-3

**Published:** 2023-04-21

**Authors:** Bryan S. Blette, Anders Granholm, Fan Li, Manu Shankar-Hari, Theis Lange, Marie Warrer Munch, Morten Hylander Møller, Anders Perner, Michael O. Harhay

**Affiliations:** 1 Department of Biostatistics, Epidemiology, and Informatics, Perelman School of Medicine, University of Pennsylvania, Philadelphia, PA USA; 2 Clinical Trials Methods and Outcomes Lab, Palliative and Advanced Illness Research (PAIR) Center, Perelman School of Medicine, University of Pennsylvania, Philadelphia, PA USA; 3 Department of Intensive Care, Rigshospitalet–Copenhagen University Hospital, Copenhagen, Denmark; 4 Collaboration for Research in Intensive Care, Copenhagen, Denmark; 5 Department of Biostatistics, Yale University School of Public Health, New Haven, CT USA; 6 Center for Methods in Implementation and Prevention Science, Yale University School of Public Health, New Haven, CT USA; 7 Centre for Inflammation Research, University of Edinburgh, Edinburgh, UK; 8 Section of Biostatistics, Department of Public Health, University of Copenhagen, Copenhagen, Denmark; 9 Division of Pulmonary and Critical Care, Department of Medicine, Perelman School of Medicine, University of Pennsylvania, 304 Blockley Hall, 423 Guardian Drive, Philadelphia, PA 19104-6021 USA

**Keywords:** Risk factors, Respiratory tract diseases, Epidemiology, Outcomes research, Translational research

## Abstract

The currently recommended dose of dexamethasone for patients with severe or critical COVID-19 is 6 mg per day (mg/d) regardless of patient features and variation. However, patients with severe or critical COVID-19 are heterogenous in many ways (e.g., age, weight, comorbidities, disease severity, and immune features). Thus, it is conceivable that a standardized dosing protocol may not be optimal. We assessed treatment effect heterogeneity in the COVID STEROID 2 trial, which compared 6 mg/d to 12 mg/d, using a causal inference framework with Bayesian Additive Regression Trees, a flexible modeling method that detects interactive effects and nonlinear relationships among multiple patient characteristics simultaneously. We found that 12 mg/d of dexamethasone, relative to 6 mg/d, was probably associated with better long-term outcomes (days alive without life support and mortality after 90 days) among the entire trial population (i.e., no signals of harm), and probably more beneficial among those without diabetes mellitus, that were older, were not using IL-6 inhibitors at baseline, weighed less, or had higher level respiratory support at baseline. This adds more evidence supporting the use of 12 mg/d in practice for most patients not receiving other immunosuppressants and that additional study of dosing could potentially optimize clinical outcomes.

## Introduction

COVID-19 is a heterogeneous acute illness with a high risk of death among those who become critically ill^1^. Part of the high risk of death may be attributable to severe pulmonary inflammation and hypoxemia. The use of anti-inflammatory agents has thus been the target of several experimental studies for COVID-19, as well as of other critical illness syndromes such as sepsis and acute respiratory distress syndrome (ARDS). Corticosteroids appear to improve outcomes in patients with severe or critical COVID-19^2,3^ and are recommended in WHO guidelines^4^. The currently recommended dose of dexamethasone is 6 mg per day (mg/d, corresponding to 7.2 mg/d dexamethasone phosphate) regardless of patient features and variation^4^. However, patients with severe or critical COVID-19 are heterogenous in many ways, such as age, weight, comorbidities, disease severity, and immune features^5,6^. Thus, it is conceivable that a standardized dosing protocol, ignoring the COVID-19 illness differences, may not be optimal. The COVID STEROID 2 trial^7^, which compared 12 mg/d to 6 mg/d of intravenous dexamethasone, found an average treatment effect favoring 12 mg/d of dexamethasone on the primary outcome of the number of days alive without life support (DAWOLS) at day 28 (adjusted mean difference, 1.3 days [95% CI 0 to 2.6 days]; *P* = 0.07); day 90 (adjusted mean difference, 4.4 days [99% CI − 1.6 to 10.4 days]; *P* = 0.15); and 90-day mortality (adjusted relative risk, 0.87 [99% CI 0.70 to 1.07]* P* = 0.09). A subsequent, pre-planned, Bayesian analysis was in line with the original frequentist analysis with probabilities of benefit on DAWOLS and mortality after 90 days of 85 and 95%, respectively^8^. The results of the COVID STEROID 2 trial suggest that the recommended 6 mg/d dosing may not be optimal for all patients.

In an analysis of heterogeneity of treatment effect (HTE) published in the primary trial report, 8 predefined subgroup analyses were done, but none met the pre-selected frequentist threshold for statistical significance of P-value < 0.01^7^. However, individual subgroup HTE assessments are prone to false negatives (and positives) as they do not account for overlap and differences in other factors within each examined subgroup^9,10^. Likewise, this approach to assessing HTE does not straightforwardly translate into individualized treatment guidance as patients often have multiple overlapping and complex characteristics. In an effort to examine HTE of dexamethasone for critically ill COVID-19 in a more dynamic clinical model, we applied a machine learning methodology termed “Bayesian Additive Regression Trees” or BART to the COVID STEROID 2 trial (NCT04509973) to assess variation in treatment responses among those randomized to 12 mg/d and 6 mg/d of intravenous dexamethasone^11^.

## Methods

This is a post hoc exploratory analysis of the COVID STEROID 2 trial^7^. It was conducted according to a statistical analysis plan, which was written after the pre-planned analyses of the trial were reported, but before any of the analyses reported in this manuscript were conducted (https://osf.io/2mdqn/). This manuscript was presented according to the Strengthening the Reporting of Observational Studies in Epidemiology (STROBE) checklist^12^, with Bayesian analyses reported according to the Reporting of Bayes Used in clinical STudies (ROBUST) guideline^13^.

### Rationale for using BART methodology

HTE implies that some individuals respond differently, i.e., better or worse, than others who receive the same therapy due to differences between individuals. Most trials are designed to evaluate the average treatment effect, which is the summary of all individual effects in the trial sample (see supplementary appendix for additional technical details). Traditional HTE methods examine patient characteristics one at a time, looking to identify treatment effect differences according to individual variables. This approach is well known to be limited as it is underpowered (due to adjustment for multiple testing) and does not account for the fact that many characteristics under examination are correlated and may have synergistic effects. As a result, more complex relationships between variables that better define individuals, and thus may better inform understanding about the variations in treatment response, may be missed using conventional HTE approaches. Thus, identifying true and clinically meaningful HTE requires addressing these data and statistical modeling challenges. BART is inherently an attractive method for this task, as the algorithm automates the detection of nonlinear relationships and interactions hierarchically based on the strength of the relationships, thereby reducing researchers’ discretion when analyzing experimental data. This approach also avoids any model misspecification or bias inherent in traditional interaction test procedures. BART can also be deployed, as we do herein, within the counterfactual framework to study HTE, i.e., to estimate conditional average treatment effects given the set of covariates or potential effect modifiers^11,14,15^, and has shown superior performance to competing methods in extensive simulation studies^16,17^. These features make BART an appealing tool for trialists to explore HTE to inform future confirmatory HTE analyses in trials and hypothesis generation more broadly. Thus, this analysis used BART to evaluate the presence of multivariable HTE and estimate conditional average treatment effects among meaningful subgroups in the COVID STEROID 2 trial.

### COVID STEROID 2 trial

The COVID STEROID 2 trial^7^ was an investigator-initiated, international, parallel-group, stratified, blinded, randomized clinical trial conducted at 31 sites in 26 hospitals in Denmark, India, Sweden, and Switzerland between 27 August 2020 and 20 May 2021^7,18^. The trial was approved by the regulatory authorities and ethics committees in all participating countries.

The trial enrolled 1000 adult patients hospitalized with COVID-19 and severe hypoxemia (≥ 10 L oxygen/min, use of non-invasive ventilation (NIV), continuous use of continuous positive airway pressure (cCPAP), or invasive mechanical ventilation (IMV)). Patients were primarily excluded due to previous use of systemic corticosteroids for COVID-19 for 5 or more days, unobtainable consent, and use of higher-dose corticosteroids for other indications than COVID-19^4,17^. Patients were randomized 1:1 to dexamethasone 12 mg/d or 6 mg/d intravenously once daily for up to 10 days. Additional details are provided in the primary protocol and trial report^7,18^.

The trial protocol was approved by the Danish Medicines Agency, the ethics committee of the Capital Region of Denmark, and institutionally at each trial site. The trial was overseen by the Collaboration for Research in Intensive Care and the George Institute for Global Health. A data and safety monitoring committee oversaw the safety of the trial participants and conducted 1 planned interim analysis. Informed consent was obtained from the patients or their legal surrogates according to national regulations.

### Study outcomes

We examined two outcomes: (1) DAWOLS at day 90 (i.e., the observed number of days without the use of IMV, circulatory support, and kidney replacement therapy without assigning dead patients the worst possible value), and (2) 90-day mortality. Binary mortality outcomes were used to match the primary trial analysis; time-to-event outcomes also generally tend to be less robust for ICU trials^19^. We selected DAWOLS at day 90 in lieu of the primary outcome of the trial (DAWOLS at day 28) and to align with other analyses of the trial which sought to examine outcomes in a longer term. Both outcomes were assessed in the complete intention-to-treat (ITT) population, which was 982 after the exclusion of patients without consent for the use of their data^7^. As the sample size is fixed, there was no formal sample size calculation for this study.

### Pre-selected prognostic HTE factors

While BART is a data-driven approach that can scan for interdependent relationships among any number of factors, we only examined heterogeneity across a pre-selected set of factors deemed to be clinically relevant by the authors and members of the COVID STEROID 2 trial Management Committee. The pre-selected variables that were included in this analysis are listed below with the scale used in parentheses. Continuous covariates were standardized to have a mean of 0 and a standard deviation of 1 prior to analysis. Detailed variable definitions are available in the study protocol^18^.

1. participant age (continuous),
2. limitations in care (yes, no),
3. level of respiratory support (open system versus NIV/cCPAP versus IMV)
4. interleukin-6 (IL-6) receptor inhibitors (yes, no),
5. use of dexamethasone for up to 2 days versus use for 3 to 4 days prior to randomization,
6. participant weight (continuous),
7. diabetes mellitus (yes, no),
8. ischemic heart disease or heart failure (yes, no),
9. chronic obstructive pulmonary disease (yes, no), and,
10. immunosuppression within 3 months prior to randomization (yes, no).

### Statistical analysis

We evaluated HTE on the absolute scale (i.e., mean difference in days for the number of DAWOLS at day 90 and the risk difference for 90-day mortality). The analysis was separated into two stages^14,20–22^. In the first stage, conditional average treatment effects were estimated according to each participants’ covariates using BART models. The DAWOLS outcome was treated as a continuous variable and analyzed using standard BART, while the binary mortality outcome was analyzed using logit BART. In the second stage, a “fit-the-fit” approach was used, where the estimated conditional average treatment effects were used as dependent variables in models to identify covariate-defined subgroups’ differential treatment effects. This second stage used classification and regression trees models^23^, where the maximum depth was set to 3 as a post hoc decision to aid interpretability. As the fit-the-fit reflects estimates from the BART model, the resulting overall treatment effects (e.g., risk difference) vary slightly from the raw trial data.

BART models are often fit using a sum of 200 trees and specifying a base prior of 0.95 and a power prior of 2, which penalize substantial branch growth within each tree^15^. Although these default hyperparameters tend to work well in practice, it was possible they were not optimal for this data. Thus, the hyperparameters were evaluated using tenfold cross-validation, comparing predictive performance of the model under 27 pre-specified possibilities, namely every combination of power priors equal to 1, 2, or 3, base priors equal to 0.25, 0.5, or 0.95, and number of trees equal to 50, 200, or 400. The priors corresponding to the lowest cross-validation error were used in the final models. Each model used a Markov chain Monte Carlo procedure consisting of 4 chains that each had 100 burn-in iterations and a total length of 1100 iterations. Posterior convergence for each model was assessed using the diagnostic procedures described in Sparapani et al.^24^. Model diagnostics were good for all models. All parameters seemed to converge within the burn-in period and the z-scores for Geweke’s convergence diagnostic^25^ were approximately standard normal. All BART models were fit using R statistical computing software v. 4.1.2^26^ with the ‘BART’ package v. 2.9^24^, and all CART models were fit using the ‘rpart’ package v. 4.1.16^27^.

The analysis was performed under the ITT paradigm; compliance issues were considered minimal. As in the primary analyses of the trial, the small amount of missing outcome data was ignored in the primary analyses. Sensitivity analyses were performed under best/worst- and worst/best-case imputation. For best/worst-case imputation, the entire estimation procedure was repeated after setting all missing mortality outcome data in the 12 mg/d group to alive at 90 days and all missing mortality outcome data in the 6 mg/d group to dead at 90 days. Then, all days with missing life support data were set to alive without life support for the 12 mg/d group and the opposite for the 6 mg/d group. Under worst/best-case imputation, the estimation procedure was repeated under the opposite conditions, e.g., setting all missing mortality outcome data in the 12 mg/d group to dead at 90 days and all missing mortality outcome data in the 6 mg/d group to alive at 90 days.

The resulting decision trees from each fit-the-fit analysis described above (one for the 90-day mortality outcome, and one for the 90-day DAWOLS outcome) were outputted (with continuous variables de-standardized, i.e., back-translated to the original scales). Likewise, the resulting decision trees for each outcome after best- and worst-case imputation were outputted for comparison with the complete records analyses. All statistical code is made available at https://github.com/harhay-lab/Covid-Steroid-HTE.

## Results

### Overall effects

All 982 patients in the ITT population were analyzed. A summary of the study sample and outcomes was reported in the primary publication; summary statistics for the outcomes and effect modifiers considered in this analysis are provided in Table 1^7^. The study groups were largely similar, though the number of comorbidities was slightly lower in the 12 mg/d group driven by a lower prevalence of diabetes. The 12 mg/d group had a higher median number of DAWOLS and lower 90-day mortality: the median number of DAWOLS was 84.0 days (IQR, 9.3 to 90.0 days) in the 12 mg/d of dexamethasone group and 80.0 days (IQR, 6.0 to 90.0 days) in the 6 mg/d of dexamethasone group (adjusted mean difference, 4.4 days [99% CI − 1.6 to 10.4 days]). At 90 days, 157 of 490 patients (32.0%) had died in the 12 mg/d group and 180 of 478 patients (37.7%) had died in the 6 mg/d group (adjusted relative risk, 0.87 [99% CI 0.70–1.07]).

**Table 1:** Summary statistics for the potential effect modifiers and outcomes by treatment arm.

Characteristic/outcome	12 mg/d of dexamethasone (n = 497)	6 mg/d of dexamethasone (n = 485)
Potential baseline effect modifiers		
Age, median (IQR), years	65 (56, 74)	64 (54, 72)
Limitations in care, no. (%)	30 (6%)	25 (5%)
Respiratory support, no. (%)		
Open system	272 (55%)	258 (53%)
Non-invasive ventilation or continuous positive airway pressure	118 (24%)	128 (26%)
Invasive mechanical ventilation	107 (22%)	99 (20%)
Interleukin-6 receptor inhibitors, no. (%)	52 (11%)	47 (10%)
Use of dexamethasone prior to randomization, no. (%)		
Up to 2 days prior	384 (77%)	355 (73%)
3–4 days prior	113 (23%)	130 (27%)
Weight, median (IQR), kg	80 (68, 96)	80 (68, 95)
Diabetes mellitus, No. (%)	135 (27%)	163 (34%)
Ischemic heart disease or heart failure, no. (%)	67 (14%)	69 (14%)
Chronic obstructive pulmonary disease, no. (%)	57 (12%)	56 (12%)
Immunosuppression within 3 months prior to randomization, no. (%)	40 (8%)	43 (9%)
Outcomes		
Days alive without life support at 90 days, median (IQR)	84 (9, 90) [n = 489]	80 (6, 90) [n = 478]
Mortality at 90 days, no./total (%)	157/490 (32%)	180/478 (38%)

Some of these results were previously reported in Tables 1 and 2 of the primary manuscript for this trial.

mg/d, milligrams per day.

### BART results for 90-day DAWOLS outcome

**Figure 1:**
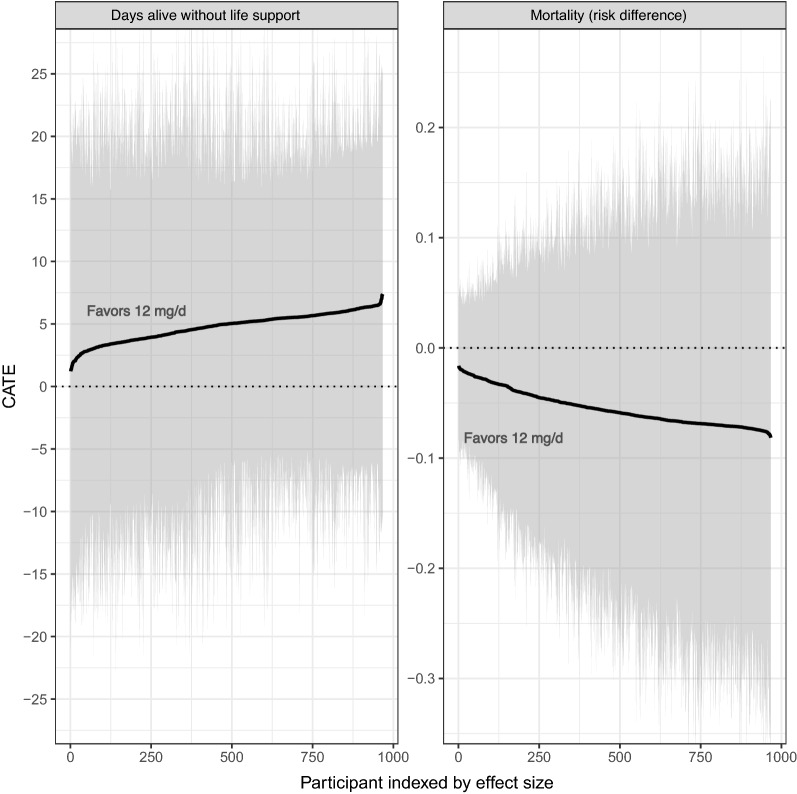
Estimated conditional average treatment effects (CATEs) for each individual in the trial. The left panel provides estimates (solid line) and 95% credible intervals (shaded area) for the difference in days without life support at 90 days, while the right panel provides estimates (solid line) and 95% credible intervals (shaded area) for the mortality risk difference.

**Figure 2:**
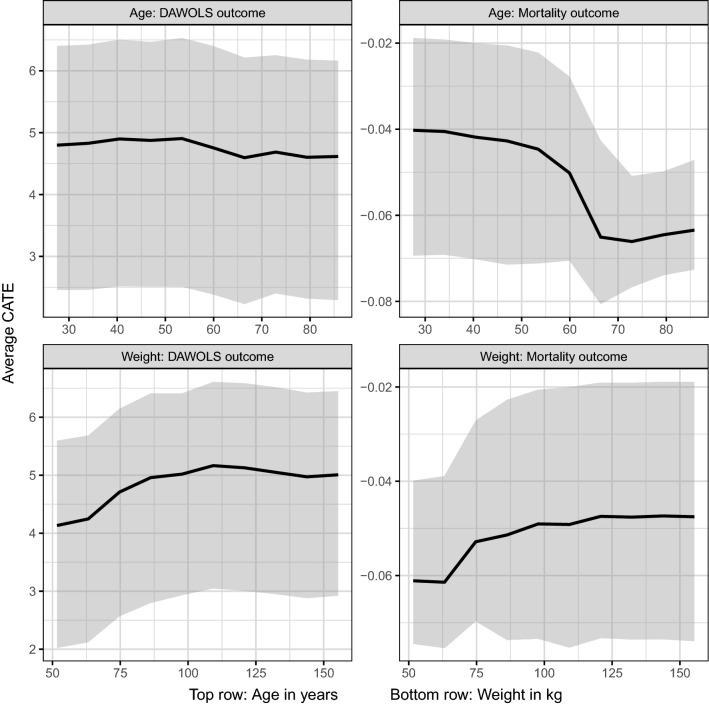
Partial dependence plots exploring how the conditional average treatment effects vary across the continuous covariates. The top row of panels displays results for the age covariate, while the bottom row displays results for the weight covariate. Within each row, the left panel displays estimates (solid line) and 95% credible intervals (shaded area) for the difference in days without life support at 90 days, while the right panel displays estimates (solid line) and 95% credible intervals (shaded area) for the mortality risk difference.

**Figure 3:**
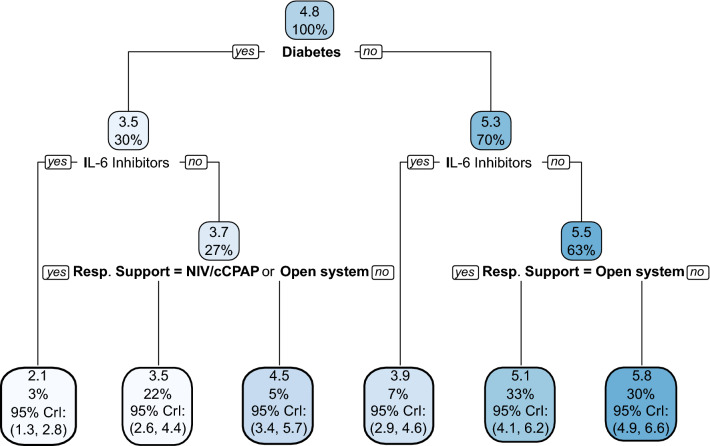
Model results for the continuous outcome days without life support at day 90. The top value in each box is the estimated mean difference in days alive without life support (12 mg/d group minus 6 mg/d group) in the subgroup with corresponding covariate values. The bottom value in each box is the proportion of the trial sample belonging to the subgroup. A complete walk through of the figure is provided in the main text (see results). The first square at the top indicates that the estimated overall mean difference in the trial was 4.8 days in favor of 12 mg/d. Next, for those with diabetes (30% of the sample), the estimated mean difference was 3.5 days, while for those without diabetes (70% of the sample) the estimated mean difference was 5.3 days, both in favor of 12 mg/d. Subsequent splits in the decision tree can be interpreted similarly. Cross validation selected a power prior of 1, a base prior of 0.95, and 400 trees.

The BART analysis generally complemented the findings reported in the average treatment effect from the original trial. Estimated conditional average treatment effects for DAWOLS at day 90 ranged from about 1 to 7 days, favoring 12 mg/d of dexamethasone (Fig. 1). Partial dependence plots showed stronger treatment effects for patients that were older and lower weight (Fig. 2). Receiving 12 mg/d of dexamethasone was associated with improved outcomes among all the identified subgroups; however, some subgroups had more substantial effects. For the DAWOLS at day 90 (Fig. 3), the decision tree initially split by diabetes status at baseline, where 12 mg/d was less beneficial for patients with diabetes. Next, for both those with and without diabetes, 12 mg/d was less beneficial for patients using IL-6 inhibitors at baseline. Finally, for those with diabetes who were not using IL-6 inhibitors, 12 mg/d was more beneficial when using IMV than NIV/cCPAP or open systems. For those without diabetes who were not using IL-6 inhibitors, 12 mg/d was more beneficial when using either IMV or NIV/cCPAP than open systems. Overall, the lowest estimated effect size was an improvement of 2.1 DAWOLS for patients with diabetes and using IL-6 inhibitors in favor of 12 mg/d. The highest estimated effect size was an improvement of 5.8 DAWOLS for patients without diabetes, not using IL-6 inhibitors, and using IMV or NIV/cCPAP in favor of 12 mg/d. There was some overlap in credible intervals across subgroups, although the groups with the lowest and highest estimated treatment effects had non-overlapping intervals. Note that the interval interpretation is a 95% credible region for the subgroup mean, and not a region where 95% of individual treatment effects within each subgroup would lie. Alternative subgroups defined by quartile of estimated treatment effect yielded similar conclusions (Table 2).

**Table 2:** Treatment effect summaries for subgroups defined by quartile of estimated treatment effect for each of (1) estimated mean difference in days alive without life support at 90 days and (2) estimated mortality risk difference.

Group	Posterior mean DAWOLS 12 mg/d	Posterior mean DAWOLS 6 mg/d	DAWOLS mean CATE (95% CrI)	Posterior mortality risk 12 mg/d	Posterior mortality risk 6 mg/d	Mortality mean CATE (95% CrI)
Q1: CATE	59.8	56.6	3.2 (1.9, 3.9)	13.0%	16.3%	− 3.3 (− 4.4, − 2.0)
Q2: CATE	58.4	53.9	4.5 (3.9, 5.0)	26.7%	31.9%	− 5.2 (− 5.8, − 4.5)
Q3: CATE	60.3	55.0	5.3 (5.0, 5.6)	39.5%	45.9%	− 6.4 (− 6.8, − 5.8)
Q4: CATE	58.4	52.3	6.1 (5.6, 6.7)	45.3%	52.4%	− 7.2 (− 7.8, − 6.8)

To briefly summarize, this table complements Fig. 1 and shows that 12 mg/d is associated with greater benefit for both outcomes compared to 6 mg/d. Figure 1 shows the results for each individual, while this table summarizes across quartiles of the distribution. CATE, conditional average treatment effect; CrI, credible interval; DAWOLS, days alive without life support; mg/d, milligrams per day.

### BART results for 90-day mortality

**Figure 4:**
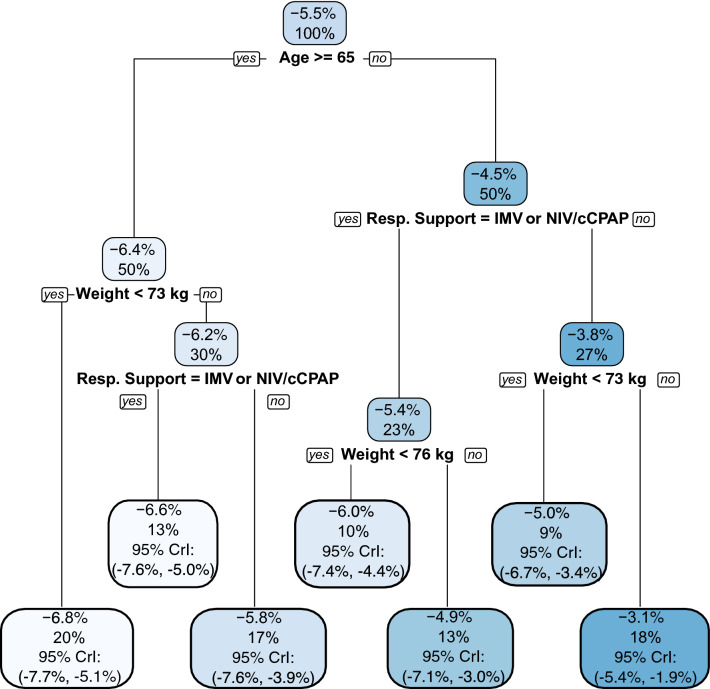
Model results for the binary outcome mortality at day 90. The top value in each box is the estimated treatment effect in the subgroup with corresponding covariate values (risk difference for 12 mg/d vs. 6 mg/d). The bottom value in each box is the proportion of the trial sample belonging to the subgroup. A complete walk through of the figure is provided in the main text (see results). The first square at the top indicates that the overall estimated risk difference in the trial was 5.5% in favor of 12 mg. Next, for those aged 65 years or older (50% of the sample), the estimated risk difference was 6.4%, while for those under age 65 (50% of the sample) the estimated risk difference was 4.5%, both in favor of 12 mg/d. Subsequent splits in the decision tree can be interpreted similarly. Cross validation selected a power prior of 3, a base prior of 0.25, and 400 trees.

For the 90-day mortality outcome, estimated conditional risk differences ranged from about 1% to 8%, favoring 12 mg/d of dexamethasone (Fig. 1). Partial dependence plots showed stronger treatment effects for patients that were lower weight, but little variation across age (Fig. 2). In the second-stage CART analysis (Fig. 4), the decision tree initially split by age, where 12 mg/d was more beneficial for patients who were at least 65 years old. Continuing with patients at least 65 years old, 12 mg/d was more beneficial for those who weighed less than 73 kg (kg). Then among patients who were older than 65 and weighed more than 73 kg, 12 mg/d was more beneficial to those using IMV or NIV/cCPAP than open systems. Next, considering patients who were less than 65 years old, 12 mg/d was more beneficial to those using IMV or NIV/cCPAP than open systems. Regardless of respiratory support, 12 mg/d was more beneficial for patients who weighed less than about 73 kg (with minor differences in the cutoff as displayed in Fig. 4). Overall, the smallest estimated risk difference was 3.1% in favor of 12 mg/d among patients who were less than 65 years old, on open systems, and weighing more than 73 kg. The largest estimated risk difference was 6.8% in favor of 12 mg/d among patients who were at least 65 years old and weighing less than 73 kg. As with the DAWOLS outcome, alternative subgroups defined by quartile of estimated treatment effect yielded similar conclusions (Table 2).

For both outcomes, the level of respiratory support was a key modifier with larger effect sizes (favoring 12 mg/d) for those on IMV, NIVN and cCPAP than those on open systems. Sensitivity analyses under best- and worst-case imputation of the missing outcomes resulted in very similar final decision trees that selected the same covariates and resulted in nearly identical subgroups and conclusions (Supplemental Figs. 1–4).

## Discussion

This exploratory analysis of the COVID STEROID 2 trial using BART provides a deeper and more clinically aligned examination of HTE that leveraged both the flexible modeling and causal inference framework that BART provides. Overall, our findings are generally in line with the primary trial results^7^. Specifically, we found consistent and relatively strong evidence of a large positive average effect of the intervention dosing of 12 mg/d across all 10 baseline measures we examined, but some subgroups had more substantial benefits. These findings supplement the conventional subgroup analyses in the primary report^7^ and the recently reported one-by-one effect modifier analysis considering HTE on the continuous scale for multiple relevant baseline variables^28^, however, this analysis has additional advantages as it considers multiple potential effect modifiers simultaneously and thus accounts for the fact that many characteristics under examination are correlated and may have synergistic effects.

Specifically, we found that the individuals who required higher level respiratory support benefited the most from 12 mg/d of dexamethasone. Second, those treated with IL-6 inhibitors at baseline had less benefit from 12 mg/d, indicating that higher doses of dexamethasone may not be needed in patients receiving IL-6 inhibitors, or that concurrent administration of a higher dose of dexamethasone alongside IL-6 blockers may be associated with adverse effects overwhelming the additional benefit. These findings are in line with previous results, indicating that the benefits of corticosteroids are greater for patients on IMV than patients on oxygen only^2^. Critically ill COVID-19 patients requiring IMV have greater systemic and pulmonary inflammation, alongside dysregulated immune responses^29^, with distinct COVID-19 immune response subpopulations (i.e., subphenotypes)^30–32^. Corticosteroids have genomic, and non-genomic effects that are dose dependent^33^. As IL-6 plays a major role in COVID-19 pathophysiology^29^, and IL-6 blockers have a treatment effect^34^, the additional treatment effect of higher doses of corticosteroids is likely to be smaller in patients receiving IL-6 inhibitors.

An interesting finding was the suggested effect modification with weight. Indeed, there was discussion after the original trial publication that motivated the inclusion of weight into this analysis^35^. Specifically, it has been proposed that the 6 mg/d dose may not be enough in patients with higher body weights, so intuitively, if this were correct, we would expect to see increased benefit with 12 mg/d in higher-weight patients. However, this is the opposite of what we observed. Some possible explanations for this are (1) that the decision tree algorithm chose a different weight cutoff than was previously explored, (2) that prior comparisons for weight subgroups focused on 28-day outcomes rather than 90-day outcomes, or (3) the output of the decision trees includes higher-level interactions, so in addition to weight, the output incorporates age and respiratory support as well. Furthermore, the partial dependence plots indicated that the weight results may have been driven by patients who were very low weight, and that treatment effects were similar across other patients.

Our work has limitations. Foremost, this was a post hoc analysis, but with a pre-specified protocol that was drafted and published before any analyses was undertaken. Second, BART is a data-driven methodology and may find small correlations and dependencies that may not be clinically relevant or actionable. To minimize this, we used a small pre-specified set of covariates for exploratory analysis and avoided a confirmatory hypothesis testing framework with multiple comparisons issues. In addition, the rationale for adopting BART is that previous simulation experiments have shown that this approach has relatively robust performance in exploring HTE in both randomized trials and observational studies^14,17,36^. Potential improvement of the current implementation of BART is possible, for example, by running Bayesian nonparametric regression on the estimated pseudo-outcome under a doubly-robust approach^37^. As the pseudo-outcome tends to be less noisy than the observed outcome, this approach may also improve the quality of the subgroup detection during the fit-the-fit step. This is a promising idea that is worth future development and evaluation but falls beyond the scope of the current article. Third, and relatedly, there may be subgroups that are vulnerable to higher dose corticosteroid use who were either not represented in selected covariates or not included in the trial sample. We did not assess potential HTE according to the number of serious adverse reactions as the event counts were smaller than for mortality^7^ and as effects on serious adverse reactions would likely, in turn, affect DAWOLS and mortality at day 90. There are also several sources of uncertainty in the analysis, including potential mild measurement error in covariates and uncertainty related to the model fitting procedure, such as the hyperparameter selection. Finally, some of the assessed baseline variables (e.g., use of IL-6 inhibitors and some comorbidities) appeared in relatively few patients in the trial^7^, which may have influenced the ability of BART to identify HTE according to these characteristics.

In summary, this exploratory analysis found some evidence of HTE, but no qualitatively different effects, i.e., we did not find benefit in some groups and harm in others. The clinical relevance of these results is that they provide additional evidence supporting the use of 12 mg/d dexamethasone in practice for most patients. However, they also suggest that additional study of dosing and consideration of some individual characteristics could potentially optimize clinical outcomes. Further, we have provided a concrete example of how to use BART to assess HTE in a clinical trial and extract information from clinical trial data to potentially inform individualized treatment decisions.

## Supplementary Information

Supplementary Information 1.

Supplementary Information 2.

## Data Availability

Deidentified patient data that supports the findings of this study will be made available to researchers by the COVID STEROID 2 Trial Management committee (contact@cric.nu) under a data access agreement.

## References

[1] Haase N (2022). Changes over time in characteristics, resource use and outcomes among ICU patients with COVID-19—A nationwide, observational study in Denmark. Acta Anaesthesiol. Scand..

[2] Group, R. C. (2021). Dexamethasone in hospitalized patients with Covid-19. N. Engl. J. Med..

[3] W. H. O. Rapid Evidence Appraisal for COVID-19 Therapies Working Group (2020). Association between administration of systemic corticosteroids and mortality among critically ill patients with COVID-19. JAMA.

[4] Agarwal A (2020). A living WHO guideline on drugs for covid-19. BMJ.

[5] Laing AG (2020). A dynamic COVID-19 immune signature includes associations with poor prognosis. Nat. Med..

[6] Mathew D (2020). Deep immune profiling of COVID-19 patients reveals distinct immunotypes with therapeutic implications. Science.

[7] Covid Steroid Trial Group (2021). Effect of 12 mg vs 6 mg of dexamethasone on the number of days alive without life support in adults with COVID-19 and severe hypoxemia: The COVID STEROID 2 randomized trial. JAMA.

[8] Granholm A (2022). Dexamethasone 12 mg versus 6 mg for patients with COVID-19 and severe hypoxaemia: A pre-planned, secondary Bayesian analysis of the COVID STEROID 2 trial. Intensive Care Med..

[9] Burke JF, Sussman JB, Kent DM, Hayward RA (2015). Three simple rules to ensure reasonably credible subgroup analyses. BMJ.

[10] Brookes ST (2001). Subgroup analyses in randomised controlled trials: Quantifying the risks of false-positives and false-negatives. Health Technol. Assess..

[11] Carnegie N, Dorie V, Hill JL (2019). Examining treatment effect heterogeneity using BART. Observ. Stud..

[12] von Elm E (2007). The Strengthening the Reporting of Observational Studies in Epidemiology (STROBE) statement: Guidelines for reporting observational studies. Ann. Intern. Med..

[13] Sung L (2005). Seven items were identified for inclusion when reporting a Bayesian analysis of a clinical study. J. Clin. Epidemiol..

[14] Hu L, Ji J, Li F (2021). Estimating heterogeneous survival treatment effect in observational data using machine learning. Stat. Med..

[15] Hill J, Linero A, Murray J (2020). Bayesian additive regression trees: A review and look forward. Annu. Rev. Stat. Appl..

[16] Dorie V, Hill J, Shalit U, Scott M, Cervone D (2019). Automated versus do-it-yourself methods for causal inference: Lessons learned from a data analysis competition. Stat. Sci..

[17] Wendling T (2018). Comparing methods for estimation of heterogeneous treatment effects using observational data from health care databases. Stat. Med..

[18] Munch MW (2021). Higher vs lower doses of dexamethasone in patients with COVID-19 and severe hypoxia (COVID STEROID 2) trial: Protocol and statistical analysis plan. Acta Anaesthesiol. Scand..

[19] Schoenfeld D (2005). Survival methods, including those using competing risk analysis, are not appropriate for intensive care unit outcome studies. Crit. Care.

[20] Logan BR, Sparapani R, McCulloch RE, Laud PW (2019). Decision making and uncertainty quantification for individualized treatments using Bayesian Additive Regression Trees. Stat. Methods Med. Res..

[21] Lu M, Sadiq S, Feaster DJ, Ishwaran H (2018). Estimating individual treatment effect in observational data using random forest methods. J. Comput. Graph Stat..

[22] Woody S, Carvalho CM, Murray JS (2021). Model interpretation through lower-dimensional posterior summarization. J. Comput. Graph. Stat..

[23] Breiman L, Friedman JH, Olshen RA, Stone CJ (2017). Classification and Regression Trees.

[24] Sparapani R, Spanbauer C, McCulloch R (2021). Nonparametric machine learning and efficient computation with Bayesian additive regression trees: The BART R package. J. Stat. Softw..

[25] Geweke JF (1991). Evaluating the Accuracy of Sampling-Based Approaches to the Calculation of Posterior Moments.

[26] R: A language and environment for statistical computing (R Foundation for Statistical Computing, 2021).

[27] rpart: Recursive Partitioning and Regression Trees. R package version 4.1-15. https://CRAN.R-project.org/package=rpart (2019).

[28] Granholm A (2022). Heterogenous treatment effects of dexamethasone 12 mg vs. 6 mg in patients with COVID-19 and severe hypoxaemia—post hoc exploratory analyses of the COVID STEROID 2 trial. Acta Anaesthesiol. Scand..

[29] van de Veerdonk FL (2022). A guide to immunotherapy for COVID-19. Nat. Med..

[30] Fish M (2022). Coronavirus disease 2019 subphenotypes and differential treatment response to convalescent plasma in critically ill adults: Secondary analyses of a randomized clinical trial. Intensive Care Med..

[31] Grant RA (2021). Circuits between infected macrophages and T cells in SARS-CoV-2 pneumonia. Nature.

[32] Lucas C (2020). Longitudinal analyses reveal immunological misfiring in severe COVID-19. Nature.

[33] Panettieri RA (2019). Non-genomic effects of glucocorticoids: An updated view. Trends Pharmacol. Sci..

[34] W. H. O. Rapid Evidence Appraisal for COVID-19 Therapies Working Group (2021). Association between administration of IL-6 antagonists and mortality among patients hospitalized for COVID-19. JAMA.

[35] Munch MW, Granholm A, Perner A (2022). Dexamethasone and number of days alive without life support in adults with COVID-19 and severe hypoxemia-reply. JAMA.

[36] Spanbauer C, Sparapani R (2021). Nonparametric machine learning for precision medicine with longitudinal clinical trials and Bayesian additive regression trees with mixed models. Stat. Med..

[37] Kennedy, E. H. Towards optimal doubly robust estimation of heterogeneous causal effects. *arXiv preprint *arXiv:2004.14497 (2020).

[38] Munch MW (2021). Low-dose hydrocortisone in patients with COVID-19 and severe hypoxia: The COVID STEROID randomised, placebo-controlled trial. Acta Anaesthesiol. Scand..

