# Supplementary material for: Causal Bayesian machine learning to assess treatment effect heterogeneity by dexamethasone dose for patients with COVID-19 and severe hypoxemia
Source: Sci Rep. 2023 Apr 21;13:6570. doi: 10.1038/s41598-023-33425-3 (PMC10120498; doi:10.1038/s41598-023-33425-3)

Supplementary Appendix
for
Causal Bayesian machine learning to assess treatment effect heterogeneity by dexamethasone dose for patients with COVID-19 and severe hypoxemia

Bryan S. Blette*[1,2], Anders Granholm*[3,4], Fan Li[5,6], Manu Shankar-Hari[7], Theis Lange[8], Marie Warrer Munch[3,4], Morten Hylander Møller[3,4], Anders Perner[3,4], and Michael O. Harhay**[1,2,9]

*Indicates co-first authors
**Indicates corresponding author, email: mharhay@pennmedicine.upenn.edu
[1]Department of Biostatistics, Epidemiology, and Informatics, Perelman School of Medicine, University of Pennsylvania, Pennsylvania, USA
[2]Clinical Trials Methods and Outcomes Lab, Palliative and Advanced Illness Research (PAIR) Center, Perelman School of Medicine, University of Pennsylvania, Pennsylvania, USA
[3]Department of Intensive Care, Rigshospitalet – Copenhagen University Hospital, Copenhagen, Denmark
[4]Collaboration for Research in Intensive Care, Copenhagen, Denmark
[5]Department of Biostatistics, Yale University School of Public Health, New Haven, Connecticut USA
[6]Center for Methods in Implementation and Prevention Science, Yale University School of Public Health, New Haven, CT, USA
[7]Centre for Inflammation Research, University of Edinburgh, Edinburgh, United Kingdom
[8]Section of Biostatistics, Department of Public Health, University of Copenhagen, Copenhagen, Denmark
[9]Division of Pulmonary and Critical Care, Department of Medicine, Perelman School of Medicine, University of Pennsylvania, Pennsylvania, USA

October 2022

Contents

1 Extended Methods **3**

1.1 Rationale for using the Bayesian Additive Regression Trees (BART) methodology 3

1.2 Causal inference framework 3

1.3 Technical details: BART 4

1.4 Technical details: Second stage CART 5

2 References **6**

3 Supplementary Figures **7**

3.1 Figure E1. Model results for the continuous days without life support at day 90 outcome after best-worst imputation 7

3.2 Figure E2. Model results for the binary 90-day mortality outcome after best-worst imputation. 8

3.3 Figure E3. Model results for the continuous days without life support at day 90 outcome after worst-best imputation. 9

3.4 Figure E4. Model results for the binary 90-day mortality outcome after worst-best imputation. 10

1 Extended Methods

1.1 Rationale for using the Bayesian Additive Regression Trees (BART) methodology

Heterogeneity of treatment effect (HTE) implies that some individuals respond differently, i.e., better or worse, than others who receive the same therapy due to differences in baseline risk factors between individuals. Most randomized trials are designed to evaluate the average treatment effect (ATE), which is a summary of all individual treatment effects in the trial sample. Traditional HTE methods examine patient characteristics one at a time, looking to identify treatment effect differences according to individual baseline variables. This approach is known to be limited as it is under-powered (due to need for multiple testing adjustment) and does not account for the fact that many characteristics under examination are correlated, and may have synergistic effects in moderating the treatment benefit (or harm). As a result, more complex relationships between variables that better define individuals and thus may better inform understanding about the variations in treatment response may be missed using conventional HTE approaches. Thus, identifying true and clinically meaningful HTE requires addressing these data and statistical modeling challenges. BART is inherently an attractive method for this task, as the algorithm automates the detection of nonlinear relationships and interactions hierarchically based on the strength of the relationships, thereby reducing researchers' discretion when analyzing experimental data. That is, the flexible modeling of the outcomes using BART will avoid any model misspecification or bias inherent in traditional interaction test procedures. BART can also be deployed within the counterfactual framework to study HTE, i.e., to estimate conditional average treatment effects (CATE) given the set of covariates or potential effect modifiers. The extent to which CATE varies across individuals in the trial sample can quantify the degree of heterogeneity of treatment effects.

1.2 Causal inference framework

The goal of a trial is to estimate the effect of an intervention, denoted as Z, on an outcome, Y. Under the counterfactual outcome framework, we assume individual i has two potential outcomes: the outcome $Y_i(1)$ we would observe under intervention ($Z = 1$), and the outcome $Y_i(0)$ we would observe under control ($Z = 0$). Treatment Z has a causal effect for participant i if $Y_i(1) \neq Y_i(0)$, (i.e., the potential outcomes differ under intervention vs. control). Since individual i cannot experience both potential outcomes, and only one of the two potential outcomes can be observed for each individual in a specific trial, we cannot calculate the causal effect for participant i with the observed data. However, randomization allows estimation of the ATE ($\Delta_{ATE} = \mathrm{E}\left[Y_i\left(1\right) - Y_i\left(0\right)\right]$) across individuals without confounding. Beyond an overall summary, the treatment effect measure for each specific participant with baseline covariate vector $\mathbf{x}$ (the CATEs) are also estimable from the observed data, $\Delta_{CATE}(\mathbf{x}) = \mathrm{E}\left[Y_i\left(1\right) - Y_i\left(0\right)|\mathbf{X}_i = \mathbf{x}\right]$. HTE is inferred if the CATE $\Delta_{CATE}\left(\mathbf{x}_i\right) \neq \Delta_{CATE}(\mathbf{x}_j)$ for two different covariate vectors.

1.3 Technical details: BART

In this paper we used a BART estimator to estimate the CATEs nonparametrically (Chen et al. 2022). Introduced by Chipman et al. (2010) and advanced by Hill (2011), BART is an ensemble method in which the mean function of a regression is approximated by the sum of individual trees, with prior distributions imposed to regularize the fit by keeping the individual tree effects to be relatively small. Notation is borrowed from these papers to illustrate the method. Specifically, let $\mathcal{T}$ denote a binary tree consisting of a set of interior node decision rules and a set of terminal nodes. Let $\mathcal{M} = \{\mu_1, \mu_2, ..., \mu_b\}$ denote a set of parameter values associated with each of the b terminal nodes of $\mathcal{T}$. The mean function with BART relies on a collection of J binary trees $\{\mathcal{T}_1, ..., \mathcal{T}_J\}$ and their corresponding set of terminal node values $\{\mathcal{M}_1, ..., \mathcal{M}_J\}$ for each binary tree, where $\mathcal{M}_j = \{\mu_{j1}, \mu_{j2}, ..., \mu_{jb_j}\}$. Each tree $\mathcal{T}_j$ consists of a sequence of decision rules through which any covariate vector can be assigned to one terminal node of $\mathcal{T}_j$ by following the decision rules prescribed at each of the interior nodes: i.e., each binary tree generates a partition of the predictor space where each element $\mathbf{X}$ of the predictor space belongs to exactly one of the b_j terminal nodes of $\mathcal{T}_j$. The decision rules at the interior nodes of $\mathcal{T}_j$ are of the form $\{X_k \leq c\}$ versus $\{X_k > c\}$, where X_k denotes the kth element of $\mathbf{X}$. A covariate $\mathbf{X}$ that corresponds to the lth terminal node of $\mathcal{T}_j$ is assigned the value μ_{jl} and $h(\mathbf{X}; \mathcal{T}_j, \mathcal{M}_j)$ denotes the function returning $\mu_{jl} \in \mathcal{M}_j$ whenever $\mathbf{X}$ is assigned to the lth terminal node of $\mathcal{T}_j$. The mean function of a generic regression model, $m(\mathbf{X})$, can then be represented as a sum of individual trees

$$m(\mathbf{X}) = \sum_{j=1}^{J} h(\mathbf{X}; \mathcal{T}_j, \mathcal{M}_j)$$

. Under the BART formulation, the trees $\mathcal{T}_j$ and node values $\mathcal{M}_j$ can be thought of as model parameters. The prior distributions on these parameters induce a prior on $h(\mathbf{X}; \mathcal{T}_j, \mathcal{M}_j)$ which induces a prior on the mean function $m(\mathbf{X})$. One needs to specify the following to completely specify the prior on $(\mathcal{T}_1, \mathcal{M}_1), \ldots, (\mathcal{T}_J, \mathcal{M}_J)$:

(i) the distribution on the choice of splitting variable at each internal node;

(ii) the distribution of the splitting value c used at each internal node;

(iii) the probability that a node at a given node-depth δ splits, which is assumed to be equal to $\tau(1 + \delta)^{-\gamma}$; and

(iv) the distribution of the terminal node values μ_{jl}.

For (i) - (iii), we used defaults suggested in Chipman et al. (2010), where, for (i), the splitting variable is chosen uniformly from the set of available splitting variables at each interior node; for (ii), a uniform prior on the discrete set of available splitting values is adopted; for (iii), the depth-related hyperparameters are chosen from a pre-specified set via 10-fold cross-validation, as described in the main paper. For (iv), the distribution of the terminal node values μ_{jl} is assumed to be $\mu_{jl} \sim \mathcal{N}\{0, (4w^2J)^{-1}\}$, where w and J are determined via 10-fold cross-validation. All BART

models were fit using the `BART` package in R. All code used to perform the analysis is publicly available at https://github.com/harhay-lab/Covid-Steroid-HTE.

1.4 Technical details: Second stage CART

In the second stage of the analysis, we follow the fit-the-fit approach described by Chen et al. (2022) and use a CART model to find HTE subgroups defined by a decision tree. The inputs for the CART model are pre-specified baseline covariates that may be effect modifiers, while the outcomes are the estimated conditional average treatment effects from the first-stage BART model. Credible intervals can be found by taking relevant percentiles of the posterior means of individuals belonging to each subgroup, but we note that the Frequentist coverage properties of these intervals have not been studied in depth.

2 References

1. Chen, X., Harhay, M. O., Tong, G., & Li, F. (2022). A Bayesian machine learning approach for estimating heterogeneous survivor causal effects: Applications to a critical care trial. *arXiv preprint* arXiv:2204.06657

2. Chipman, H. A., George, E. I. and McCulloch, R. E. (2010). BART: Bayesian additive regression trees. *The Annals of Applied Statistics*, 4(1), pp.266-298.

3. Hill, J. L. (2011). Bayesian nonparametric modeling for causal inference. *Journal of Computational and Graphical Statistics*, 20(1), pp.217-240.

3 Supplementary Figures

The following supplementary figures represent results from various sensitivity analyses described in the main manuscript.

3.1 Figure E1. Model results for the continuous days without life support at day 90 outcome after best-worst imputation

The top value in each box is the estimated mean difference in days alive without life support (12 mg minus 6 mg) in the subgroup with corresponding covariate values. The bottom value in each box is the proportion of the trial sample belonging to the subgroup.

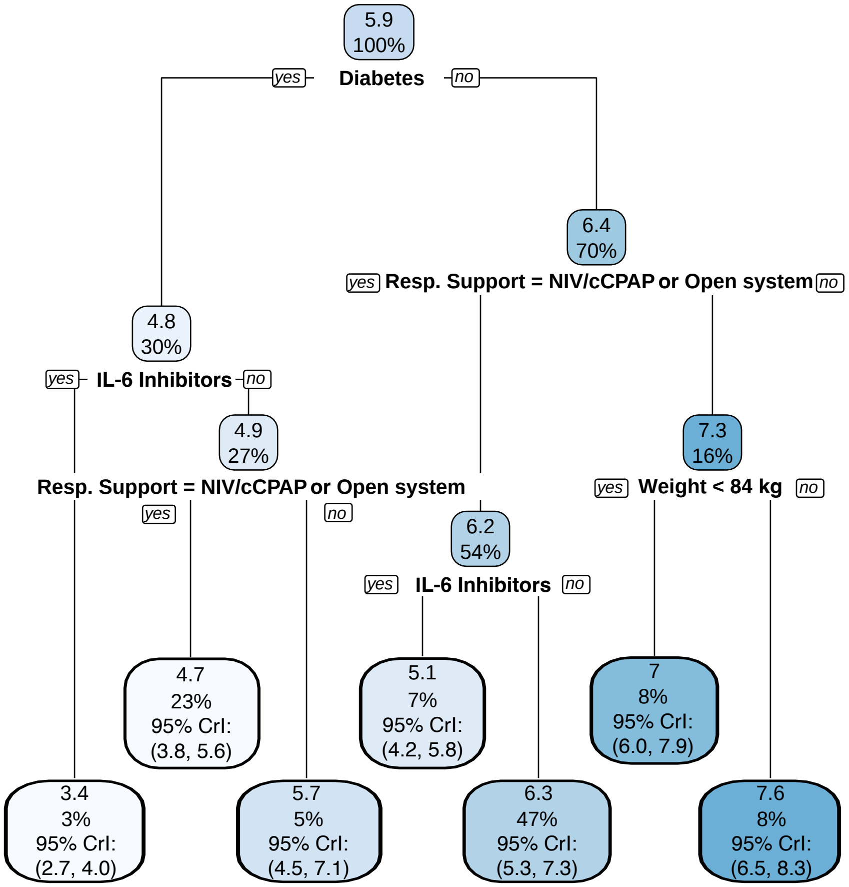

3.2 Figure E2. Model results for the binary 90-day mortality outcome after best-worst imputation.

The top value in each box is the estimated treatment effect in the subgroup with corresponding covariate values (risk difference for 12mg vs. 6mg). The bottom value in each box is the proportion of the trial sample belonging to the subgroup.

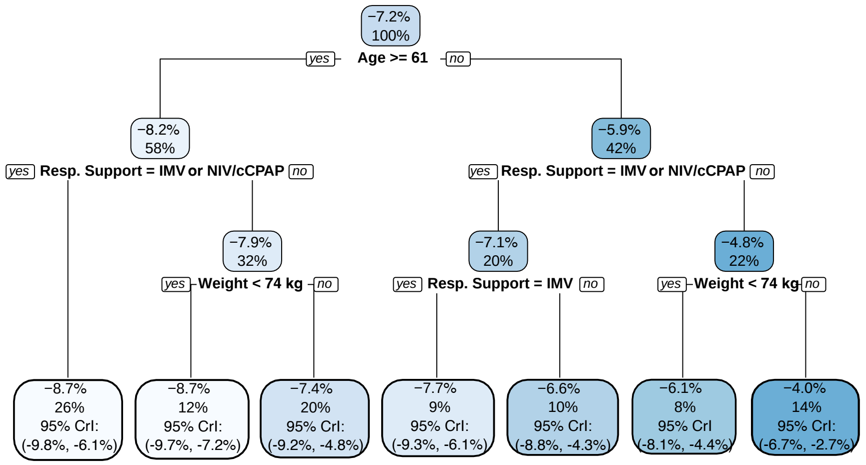

3.3 Figure E3. Model results for the continuous days without life support at day 90 outcome after worst-best imputation.

The top value in each box is the estimated mean difference in days alive without life support (12 mg minus 6 mg) in the subgroup with corresponding covariate values. The bottom value in each box is the proportion of the trial sample belonging to the subgroup.

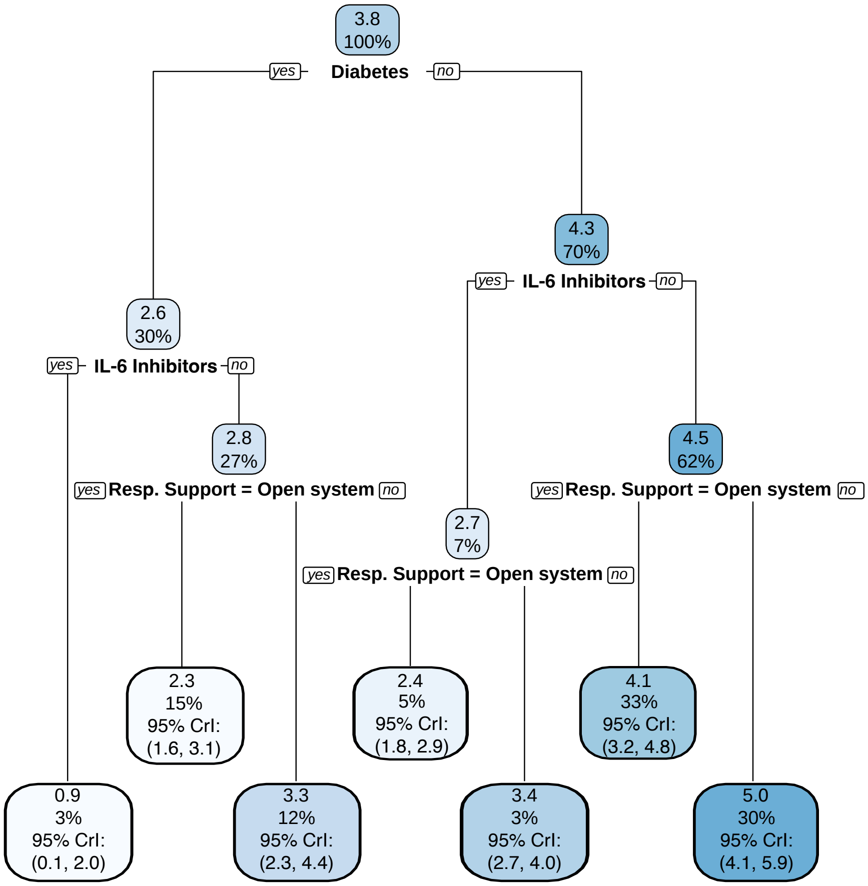

3.4 Figure E4. Model results for the binary 90-day mortality outcome after worst-best imputation.

The top value in each box is the estimated treatment effect in the subgroup with corresponding covariate values (risk difference for 12mg vs. 6mg). The bottom value in each box is the proportion of the trial sample belonging to the subgroup.

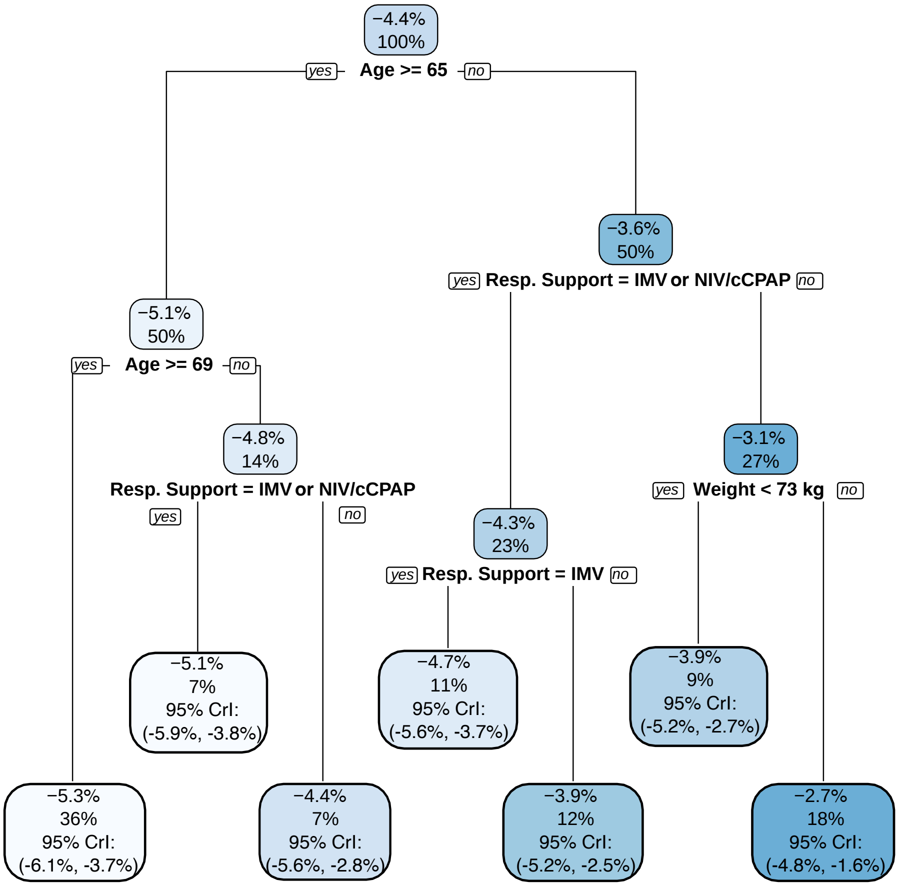

Supplement: Supplementary file 1 — Supplementary Information 1. [file 41598_2023_33425_MOESM1_ESM.pdf]
